# Alpha-synuclein spreading in Parkinson’s disease

**DOI:** 10.3389/fnana.2014.00159

**Published:** 2014-12-18

**Authors:** Ariadna Recasens, Benjamin Dehay

**Affiliations:** ^1^Neurodegenerative Diseases Research Group, Vall d’Hebron Research Institute – Center for Networked Biomedical Research on Neurodegenerative DiseasesBarcelona, Spain; ^2^Institut des Maladies Neurodégénératives, Université de Bordeaux, UMR 5293Bordeaux, France; ^3^Institut des Maladies Neurodégénératives, Centre National de la Recherche Scientifique, UMR 5293Bordeaux, France

**Keywords:** α-synuclein, spreading, aggregation, Parkinson disease, neurodegenerative diseases

## Abstract

Formation and accumulation of misfolded protein aggregates are a central hallmark of several neurodegenerative diseases. In Parkinson’s disease (PD), the aggregation-prone protein alpha-synuclein (α-syn) is the culprit. In the past few years, another piece of the puzzle has been added with data suggesting that α-syn may self-propagate, thereby contributing to the progression and extension of PD. Of particular importance, it was the seminal observation of Lewy bodies (LB), a histopathological signature of PD, in grafted fetal dopaminergic neurons in the striatum of PD patients. Consequently, these findings were a conceptual breakthrough, generating the “host to graft transmission” hypothesis, also called the “prion-like hypothesis.” Several *in vitro* and *in vivo* studies suggest that α-syn can undergo a toxic templated conformational change, spread from cell to cell and from region to region, and initiate the formation of “LB–like aggregates,” contributing to the PD pathogenesis. Here, we will review and discuss the current knowledge for such a putative mechanism on the prion-like nature of α-syn, and discuss about the proper use of the term prion-like.

## INTRODUCTION

Parkinson’s disease (PD) is a common neurodegenerative disorder of unknown origin mainly characterized by the loss of neuromelanin-containing dopaminergic neurons in the substantia nigra pars compacta (SN) and the presence of intraneuronal proteinaceous cytoplasmic inclusions called Lewy bodies (LB). One of the main protein components of the LB is the protein α-synuclein (α-syn). Accompanying LB (which are located in neuronal perikarya), gross distrophic neurites containing α-syn and ubiquitin inclusions and called Lewy neurites (LN) are common in PD pathology. Besides SN dopaminergic neurons, a significant number of other central and peripheral neuronal populations exhibit Lewy pathology (combination of LB and LN), phenotypic dysregulation, or degeneration in PD patients (Dickson, 2012).

## UPDATE ON α-SYNUCLEIN AND PD

α-synuclein is a 14 kDa protein consisting of 140 amino acids which is localized to presynaptic terminals and the nucleus (Maroteaux et al., 1988), cytosol and in some cellular membranes, such as the mitochondria-associated membrane in the endoplasmic reticulum (ER; Guardia-Laguarta et al., 2014). To date, six different missense mutations – p.A53T, p.A30P, p.E64K, p.H50Q, p.G51D, p.A53E – in the gene encoding for α-syn (*SNCA)* have been identified to cause autosomal-dominant forms of PD (Polymeropoulos et al., 1997; Kruger et al., 1998; Athanassiadou et al., 1999; Spira et al., 2001; Zarranz et al., 2004; Ki et al., 2007; Choi et al., 2008; Puschmann et al., 2009; Appel-Cresswell et al., 2013; Lesage et al., 2013). Although the exact function of α-syn remains unknown, substantial evidence suggest that α-syn function is related to its capacity to interact directly with membrane phospholipids, particularly highly curved membranes such as vesicles. In particular, α-syn seems to play a role in the vesicle trafficking during the neurotransmission release.

In aqueous solution α-syn does not have a defined structure and is normally referred as a natively unfolded protein. However, the α-syn protein adopts oligomeric and/or fibrillar conformations in certain pathological conditions (such as mutations in the SNCA gene, oxidative stress and post-translational modifications). Mounting evidence suggests that the pathological α-syn species include the post-translationally modified, mutant, oligomeric or aggregated forms. These pathological species may induce toxicity by several mechanism such as (i) disrupting the normal function of α-syn in neurotransmission release, where it may act as a negative regulator of DA release (Jenco et al., 1998; Abeliovich et al., 2000; Murphy et al., 2000; Cabin et al., 2002; Chandra et al., 2005; Larsen et al., 2006; Chen et al., 2013; DeWitt and Rhoades, 2013), (ii) impairing mitochondrial structure and complex I activity, as well as mitochondrial dynamics and mitophagy (Martin et al., 2006; Devi et al., 2008; Liu et al., 2009; Chinta et al., 2010; Kamp et al., 2010; Loeb et al., 2010; Nakamura et al., 2011), (iii) disrupting ER-Golgi vesicular transport, which results in toxic ER stress (Cooper et al., 2006; Gitler et al., 2008; Thayanidhi et al., 2010) and (iv) impairing the efficiency of some protein-degradation mechanisms (Martinez-Vicente and Vila, 2013), thereby interfering with the normal physiology of the cell, and eventually leading to cell injury and death. However, it is worth noting that two recent studies contend that some α-syn oligomers may also serve an important physiological function as synaptic vesicle wranglers (Burre et al., 2014; Wang et al., 2014).

The notion that α-syn in PD may self-propagate and spread progressively between interconnected brain regions via a cell-to-cell transmission mechanism has been strongly promoted recently (**Table 1**). Braak et al. (2003) described the presence of pathological α-syn aggregates in different brain regions, such as caudal raphe nuclei, coeruleus–subcoeruleus complex and SN. Based on this finding, Braak et al. (2003) suggested the possibility that sporadic PD might progress in six stages that follow a caudo-rostral pattern. Although other groups have confirmed some of these PD stages (Bloch et al., 2006; Dickson et al., 2010; Halliday et al., 2012) not all sporadic PD cases follow this theoretical caudo-rostral pattern of progression (Burke et al., 2008; Alafuzoff et al., 2009). Moreover, this staging does not explain the absence of clinical symptoms in subjects who on autopsy have widespread α-syn pathology. Regardless of the validity of Braak staging, this model has the merit of showing that α-syn lesions in PD are not only present in the SN, but in several other brain areas including both the peripheral nervous system (PNS) and central nervous system (CNS). According to the Braak staging hypothesis, PD might originate outside of the CNS by a causative pathogen capable of entering the CNS by way of retrograde axonal and transneuronal transport, with misfolded α-syn being a possible candidate for such a pathogen. Supporting this idea, α-syn pathology is abundant in the peripheral autonomic nervous system (pANS) of patients with LB diseases (Gelpi et al., 2014). Interestingly, epicardial fat tissue obtained during cardiac surgery from patients without parkinsonism but with some premotor symptoms such as constipation and acting dreams, exhibited α-syn pathology (Navarro-Otano et al., 2013).

**Table 1:** Summary of *in vivo* studies representing the major milestones in the α-synuclein-injected toxicity.

Inoculum	Injection site	Recipients	Reference
**Central nervous system**
Symptomatic Tg M83 mice brain lysates	n.s.	Tg M83^+/+^ mice	Mougenot et al. (2012)
Recombinant mouse α-syn Symptomatic Tg M83 mice brain lysates	Striatum	C57BL/6 J mice	Luk et al. (2012a)
Recombinant human α-syn Symptomatic Tg M83 mice brain lysates	Cortex Striatum	Tg M83^+/+^ mice	Luk et al. (2012b)
Recombinant human and mouse α-syn Symptomatic Tg M83 mice brain lysates Insoluble fraction of DLB brains	SN	C57BL/6 J mice	Masuda-Suzukake et al. (2013)
Brain homogenates from Tg M83^+/+^ Human brain homogenates from MSA patients	Parietal lobe	Tg (M83^+/-^:GFAP-luc) mice	Watts et al. (2013)
Recombinant human and mouse α-syn	SN Striatum Ent. Cortex	C57BL/6 J mice	Masuda-Suzukake et al. (2014)
LB-purified from PD patients	SN Striatum	C57BL/6 J mice Non-human primates	Recasens et al. (2014)
**Peripheral Nervous Sytem**
rAAV expressing human α-syn	Left vagus nerve	Rats WT	Ulusoy et al. (2013)
Recombinant human α-syn Human SN lysates from PD patient	Intestinal wall	Rats WT	Holmqvist et al. (2014)
Recombinant human α-syn	Olfactory bulb	C57BL/6J mice	Rey et al. (2013)
Human and mouse recombinant α-syn	Hindlimb muscle	Tg M83^+/+^ mice M20 WT mice	Sacino et al. (2014b)

*α-syn, α-synuclein; DLB, dementia with Lewy body; Ent. Cortex, entorhinal cortex; GFAP, Glial fibrillary acidic protein; LB, Lewy body; Luc, luciferase; MSA, multiple system atrophy; n.s., not specified; PD, Parkinson’s disease; SN, substantia nigra; rAAV, recombinant adeno-associated virus; Str, striatum; Tg, transgenic; WT, wild-type*.

Soon after Braak’s hypothesis, two groups independently reported that embryonic mesencephalic neurons grafted into the striatum of PD patients develop LB many years after grafting (Kordower et al., 2008; Li et al., 2008) suggesting a host-to-graft transmission of the LB pathology in the human brain. Following these findings, the terms “prion” and “prion-like” started being widely used to describe the potential pathogenic mechanism of the α-syn protein. In this scenario, α-syn could be released by living cells (via an active process such as exocytosis), or by dying cells into the surrounding extracellular milieu. Thereafter, grafted neurons could take up this released α-syn through different pathways, including endocytosis. Once inside the grafted neurons, the exogenous α-syn could act as a template that promotes misfolding of endogenously produced α-syn, ultimately leading to the formation of LB (Brundin et al., 2008). However, we believed that there are still few unsolved questions that should be answered before using confidently the term “prion” to describe the α-synuclein protein (**Table 2**). In this way, it is worth noting that a third group reported no LB pathology in a patient 14-year after graft transplantation (Mendez et al., 2008). The differences between the LB presence or not in the grafts could be associated with differences in the histology protocols used, the graft environment, the years post-grafting and/or individual differences between PD patients (Brundin et al., 2008).

**Table 2:** Missing evidences or open questions about α-synuclein spreading in PD.

Open questions
What is the composition and structure of recombinant α-syn seeds, brain homogenates samples or LB-purified samples?
What are the α-syn species responsible for toxicity and spreading in recombinant α-syn seeds, brain homogenates samples or LB-purified samples?
Are there differences in biophysical or structural properties between α-syn species responsible for toxicity and spreading?
Does spreading implies infectivity?
Are α-syn species specific from a synucleinopathy to another? Is there a strain notion?
Are cofactors (intracellular or extracellular) necessary for self-propagation?
What is the contribution of the axonal transport in the spreading process?
Is glia involved in propagation to interconnected brain structures?
Is there a common pathway/pattern for tissue migration?
What is the mechanism of cell death in those α-syn spreading based models? Does the immune response play a role?
How to improve the reproducibility of recombinant α-syn seeds? α-syn assembly by PMCA or qRT-QuIC might overcome this obstacle.
Can we extrapolate the results obtained in α-syn spreading based models into human diseases?
Does the other neurodegenerative-associated proteins (Aβ, tau, huntingtin …) share the same spreading-toxic properties of α-syn?

*α-syn, α-synuclein; Aß, amyloid-beta; LB, Lewy body; PMCA, protein misfolding cyclic amplification; qRT-QuIC, quantitative real-time quaking-induced conversion*.

## CELL-TO-CELL TRANSMISSION OF α-SYNUCLEIN

All these previous histopathological findings in human samples suggested the transmission of α-syn between cells. The question remains “can α-syn really be secreted and internalized by cells?” Since α-syn lacks an ER signal sequence that would direct it to secretory pathways, it was initially though that α-syn was exclusively an intracellular protein. However, the finding that α-syn species (monomeric and oligomeric) can be detected in human plasma and cerebrospinal fluid (CSF; Borghi et al., 2000; El-Agnaf et al., 2003) suggested the idea that α-syn can be secreted. Currently, it is well known that α-syn can be secreted into the culture medium by several types of neuronal cells (El-Agnaf et al., 2003; Lee et al., 2005; Sung et al., 2005; Emmanouilidou et al., 2010; Danzer et al., 2011). Although the exact mechanism of α-syn release has not been fully elucidated, recent results point toward a non-classic secretory pathway. In particular, it seems that α-syn may be released by exosomes in a calcium-dependent manner (Lee et al., 2005; Emmanouilidou et al., 2010) and further exacerbated after lysosomal inhibition (Alvarez-Erviti et al., 2011b).

On the other hand, several studies demonstrated that α-syn can be internalized by cells (Sung et al., 2001; Zhang et al., 2005; Danzer et al., 2007, 2009; Luk et al., 2009; Nonaka et al., 2010; Waxman and Giasson, 2010), probably by a classical endocytic mechanism (Sung et al., 2001; Lee et al., 2008a; Hansen et al., 2011; Volpicelli-Daley et al., 2011) that could include dynamin-dependent receptor-mediated endocytosis (Desplats et al., 2009; Hansen et al., 2011). However, considering the size of α-syn fibrillar aggregates, receptor-mediated endocytosis, which requires specific interactions between ligands and cell-surface receptors, seems unlikely to be the principal mode of fibril internalization. Other mechanisms could potentially mediate the transcellular movement of cytosolic α-syn aggregates [e.g., tunnel-like structures connecting two cells, called nanotubes (Gousset et al., 2009)], although these have not been fully demonstrated. Finally, α-syn monomers could potentially enter cells via passive diffusion by interacting with membranes and lipids (Ahn et al., 2006; Lee et al., 2008a; Auluck et al., 2010).

Recently, *in vitro* studies demonstrated that synthetic recombinant preformed α-syn fibrils (PFFs) could act as a seed to induce the recruitment of endogenous soluble α-syn into insoluble pathologic aggregates in cells overexpressing α-syn (Luk et al., 2009; Hansen et al., 2011; Volpicelli-Daley et al., 2011). The formation of these α-syn aggregates within recipient cells leads to alterations in synaptic functions, compromising neuronal excitability and connectivity, and culminates in neuronal death.

One of the first *in vivo* studies demonstrating that α-syn can be spread via a cell-to-cell transmission mechanism was by Desplats et al. (2009). GFP-labeled mouse cortical neuronal stem cells were injected into the hippocampus of transgenic mice expressing human α-syn under the control of the Thy-1 promoter. Four weeks after transplantation, 15% of the grafted cells exhibited human α-syn immunoreactivity. Interestingly, few of these cells exhibited inclusion bodies within the cytoplasm. In a separate study, 5% of fetal post-mitotic dopaminergic neurons grafted into the striatum of mice overexpressing human α-syn, exhibited human α-syn immunoreactivity 6 months after transplantation (Hansen et al., 2011), thus confirming the transfer of human α-syn from host-to-graft *in vivo*. In addition, this study also demonstrated that different forms of human α-syn, including monomers, oligomers and fibrils, could be taken up by neurons *in vivo* by endocytosis (Hansen et al., 2011). In addition, host-to-graft transmission of human α-syn has also been reported in rats (Kordower et al., 2011).

Once demonstrated that α-syn could be transmitted between cells, the next step was to explore the potential pathogenic effect of α-syn transmission *in vivo*. In this context, both synthetic and murine disease-associated forms of α-syn were able to induce a PD-like α-syn pathology *in vivo* (Luk et al., 2012b). Luk and colleagues reported that the intracerebral injection of brain homogenates derived from old α-syn transgenic mice (which exhibited α-syn pathology) into the neocortex and striatum of young asymptomatic transgenic mice induced a widespread accumulation of pathological α-syn throughout the anterior/posterior extent of the neural axis spanning the CNS, from olfactory bulb (OB) to the spinal cord. These effects were mostly observed by 90 days post-injection, although at 30 days post-injection some α-syn pathology was already evident. Similar results were obtained after the injection of synthetic recombinant α-syn PFFs, providing the first evidence that PFFs alone were sufficient to initiate and propagate the α-syn pathology *in vivo*. Furthermore, the inoculation of either symptomatic brain lysates or α-syn PFFs accelerated and increased the accumulation of α-syn in these transgenic mice and reduced their lifespan. Mougenot et al. (2012) reproduced part of these results. In this case, the injection of brain homogenates from symptomatic α-syn transgenic mice into the brains of healthy transgenic mice accelerated the characteristic clinical signs of paralysis observed in this mouse model and reduced the lifespan of injected animals. In addition, insoluble phosphorylated α-syn at Ser129 was also found in the brains of inoculated mice.

The pathological spreading of α-syn was also reported in wild-type (WT) mice (Luk et al., 2012a). The injection of synthetic recombinant α-syn PFFs into the striatum of WT mice induced a pathological time-dependent accumulation of endogenous α-syn that was associated with cell loss in the SN and impaired motor coordination. The formation of an LB/LN-like pathology in PFFs-inoculated mice occurred upstream of SN DA neuron loss, indicating that the α-syn pathology was sufficient to induce the cardinal behavioral and pathological features of sporadic PD. The injection of human and mouse PFFs directly into the SN (Masuda-Suzukake et al., 2013) or hippocampus (Sacino et al., 2014a) of WT mice also induced a time-dependent widespread accumulation of α-syn pathology, although no neuronal loss in the SN or motor impairment was found in this case. It is noteworthy that the α-syn spreading efficiency observed in different laboratories depends heavily on several factors which include the preparation of synthetic recombinant α-syn, the choice of the strain of mice (Sacino et al., 2014c) as well as the brain areas of inoculation (Masuda-Suzukake et al., 2014) and overall the possibility of a species barrier. Furthermore, Sacino et al. (2014c) raised an important point about the non-specific immunohistochemical staining of the Ser129-phosphorylated α-syn antibody (mAB81A). This antibody reacts with phosphor-Ser129 but also with phosphorylated neurofilament subunit L (NFL). To overcome this obstacle, this antibody has to be used cautiously associated with an optimizing protocol including (i) the use of very low antibody concentrations for minimal background; (ii) the confirmation with other phosphor-Ser129 α-syn specific antibodies and amyloid dyes such as Thioflavine S; and (iii) the combination with biochemical procedures to separate the proteins by size to detect phosphorylated α-syn.

More recently, Recasens et al. (2014) demonstrated that human α-syn species contained in PD-derived LB are pathogenic and have the capacity to initiate a PD-like pathological process, not only in rodents but also in non-human primates. Nigral LB containing pathological α-syn were purified from postmortem PD brains by sucrose gradient fractionation and subsequently inoculated into the SN or striatum of WT mice and macaque monkeys. In both mice and monkeys, intranigral or intrastriatal inoculations of PD-derived LB extracts resulted in progressive nigrostriatal neurodegeneration starting at striatal dopaminergic terminals. In LB-injected animals, exogenous human α-syn was quickly internalized within host neurons and triggered the pathological conversion of endogenous α-syn. At the onset of LB-induced neurodegeneration, host pathological α-syn diffusely accumulated within nigral neurons and anatomically interconnected brain regions. LB-induced pathogenic effects required both human α-syn present in LB extracts and host expression of α-syn. Similarly, the injection of brain homogenates from patients with other synucleinopathies, such as dementia with Lewy bodies (DLB; Masuda-Suzukake et al., 2013) and multiple system atrophy (MSA; Watts et al., 2013), triggered α-synuclein pathology in mice. While the DLB homogenate did not induce a glial response or neuronal loss, mice injected with MSA exhibited prominent astrocytic and microglial activation and developed progressive signs of neurologic dysfunction. These contradictory results concerning human α-syn-induced neurodegeneration might be explained by differences in: (i) mouse strain (WT vs. transgenic), (ii) injection site (SN vs. parietal lobe), and (iii) sample sonication (non-sonicated vs. sonicated). A further possibility is that different α-syn strains might exist in each disease (PD, MSA, and DLB), thus explaining the differences observed after the injection of each synucleinopathy sample. Supporting this concept, distinct α-syn strains generated through repetitively seeded fibrillization *in vitro* exhibited different seeding properties both *in vitro* (Bousset et al., 2013) and *in vivo* (Guo et al., 2013).

## PERIPHERAL TRANSMISSION OF α-SYNUCLEIN PATHOLOGY TO THE BRAIN

While the studies mentioned above involved a direct intracerebral inoculation of pathological α-syn, other studies have addressed the possible transmission of α-syn pathology from the periphery to the brain. For example, recombinant adeno-associated virus (rAAV) serotype 2/6-expressing human WT α-syn has been injected into the left vagus nerve in the neck of rats (Ulusoy et al., 2013). This injection induced a strong expression of human α-syn in the medulla oblongata (MO), leading to a caudo-rostral spreading of the α-syn pathology into other interconnected brain regions, such as the pontine coeruleus-subcoeruleus complex, the dorsal raphe, the hypothalamus and the amygdala. In addition, α-syn accumulation present in the aforementioned areas was accompanied by morphological evidence of neuronal abnormalities (i.e., thread-like axons with irregularly spaced, densely labeled varicosities). Surprisingly, the transmission of α-syn did not reach the SN, and neuronal damage was not induced in this brain region for at least 18 weeks after the injection.

In another study, Brundin et al. (2008) examined if α-syn could transfer from the OB to other brain structures through neuronal connections (Rey et al., 2013). To answer this question, different molecular species (monomers, oligomers composed of soluble high molecular weight species, and fibrils) of recombinant human α-syn were injected into the OB of normal mice. The authors reported that cells in different layers of the OB (i.e., the glomerular layer, mitral cell layer and granule cell layer) readily take up recombinant monomeric and oligomeric α-syn. Fibrillar α-syn was also taken up, but to a much lesser extent within the time frame of the experiments. Soon after the injection (1.5 h and 3 h), soluble and oligomer, but not fibrillar, α-syn species were detected in several interconnected brain regions, including the anterior olfactory nucleus, the frontal cortex, the tenia tecta, the olfactory tubercle, the periform cortex, the striatum and the amygdala. At these time points, few microglial cells in the OB, anterior olfactory nucleus and frontal cortex were positive for human α-syn. α-Syn in microglial cells was present only locally, and not in other brain regions 12 h after injection into the OB. In contrast, at later time points, α-syn was extensively detected in microglial cells, suggesting that microglia might clear the human α-syn released into the extracellular space by the neurons. Recently, a study from the group of Giasson reported that *in vitro-*generated PFFs induced α-syn pathology by a single peripheral intramuscular injection of α-syn in transgenic mice, associated with robust gliosis and motor impairments (Sacino et al., 2014b).

The gastrointestinal pathway has also been extensively studied. Pan-Montojo and colleagues reported that intragastric administration of the environmental toxin rotenone induced α-syn accumulation in both the enteric nervous system (ENS) and CNS following the same pattern of progression as hypothesized by Braak (Pan-Montojo et al., 2010). Firstly, they reported α-syn accumulations in ENS neurons as soon as 1–5 months after rotenone treatment. Next, they determined whether the local effect of rotenone on the ENS could lead to alterations in the synaptically connected ANS centers in the spinal cord and brainstem [i.e., in the intermediolateral nucleus in the spinal cord (IML) and dorsal motor nucleus of the vagus (DMV)]. Both the IML and DMV exhibited accumulation and aggregation of α-syn 1.5 and 3 months after rotenone treatment, although α-syn pathology in these areas was not associated with neuronal death. Interestingly, the SN also exhibited α-syn accumulation, phosphorylation and inflammatory signs 3 months after rotenone treatment. Unlike the DMV and IML, α-syn increments in the SN were associated with neuronal loss. After intragastric rotenone administration, pesticide was not detected in the blood or brain, and no inhibition of complex I activity in muscle or brain was found, suggesting that the reported alterations in the mentioned brain regions were not due to a systemic effect of rotenone. Remarkably, the rotenone-induced α-syn pathology was specific, as only neuronal subpopulations with direct connections to the ENS showed alterations, while nearby areas (e.g., striatum, cerebellum, and cortex) remained unaffected. This specificity together with the fact that the appearance of α-syn accumulations in the SN were only detected at the last treatment time-point, raised the possibility of a direct mechanism between cells being responsible for this pattern of progression of the α-syn pathology. To confirm this hypothesis, Pan-Montojo et al. (2012) severed some of the connecting nerves between the CNS and the gut, which delayed the appearance of motor symptoms after oral rotenone treatment. This treatment also stopped the progression of α-syn pathology into the IML and DMV, and prevented cell death in the SN (Pan-Montojo et al., 2012). Recently, Holmqvist et al. (2014) have demonstrated that both human α-syn present in the SN of PD patients and distinct recombinant α-syn forms (including monomers, oligomers and fibrils) can be transported via the vagal nerve to the CNS after the injection into the intestinal wall of WT adult rats.

## α-SYNUCLEIN TRANSMISSION AND NEUROINFLAMMATION

The secretion of α-syn by neurons may not only induce toxicity once inside the cytoplasm of neighboring cells, but also in the extracellular space; this may activate glial cells and induce chronic inflammation (i.e., a common pathological feature of PD), thereby contributing to the progression of the pathology throughout the brain. Supporting this idea, glial cells (i.e., astrocytes and microglia) are able to take up and degrade synthetic recombinant α-syn aggregates even more efficiently than neurons (Lee et al., 2008b). Indeed, α-syn can be transmitted between neurons and glial cells *in vitro* (Lee et al., 2010; Alvarez-Erviti et al., 2011a). Interestingly, the exposure of neuron-derived α-syn induced an inflammatory reaction in rat primary astrocytes (Lee et al., 2010) and microglia (Zhang et al., 2005; Reynolds et al., 2008; Alvarez-Erviti et al., 2011a). The direct transfer of α-syn from neurons to astrocytes was demonstrated *in vivo* using transgenic mice overexpressing human α-syn under a neuronal promoter. In these transgenic mice, abundant human α-syn accumulation was observed not only in neurons but also in glial cells (Lee et al., 2010). Consistent with these results, recombinant α-syn oligomers and monomers injected into the neocortex of WT mice were taken up by oligodendrocytes (Reyes et al., 2014). Similarly, in rAAV-treated rats overexpressing human α-syn, embryonic oligodendrocytes grafted into the striatum were found to contain this human α-syn, thus further demonstrating the neuron-to-astrocyte transmission of α-syn (Reyes et al., 2014).

## α-SYNUCLEIN AND SIDEKICKS

Recently, several studies have provided convincing evidence that this same self-propagating mechanism of the α-syn protein may be applicable to a wide range of neurodegenerative associated proteins, including Aβ, tau, huntingtin, superoxide dismutase 1 (SOD1) and TDP-43 (see Guo and Lee, 2014, for review). Each of these proteins (i.e., recombinant proteins or contained in brain lysates) have been shown to act as a template or seed that could efficiently recruit their soluble counterparts into elongating fibrils in cultured cells and/or living animals. Recently, Cicchetti et al. (2014) described the presence of mutant huntingtin (mHtt) in tissue grafted into the brains of three patients with Huntington ‘s disease (HD) who received their transplants 9–12 years before they died. Similarly to the embryonic mesencephalic neurons grafted into the striatum of PD patients which develop LB many years after grafting, the presence of mHtt in this graft tissue could be explained by the host-to-graft transmission of the neurodegenerative-associated protein Htt. However, it is worth noting that the mHtt in this study was localized to the extracellular matrix of the transplant tissue, unlike the mHtt protein aggregates found within the non-grafted regions, which localized to the neurons and neuropil (Cicchetti et al., 2014).

## CONCLUDING REMARKS AND FUTURE DIRECTIONS

Mounting evidence suggests the concept that α-syn may be responsible for initiating and spreading the pathological process in PD. Notably, cellular and animal models developed so far based on the transmission (or spreading) properties might allow to screen therapeutic approaches against α-syn pathology (Sato et al., 2014). Of interest, a recent study using the PFFs-based model of PD demonstrated that immunotherapy with antibodies specifically targeting misfolded α-syn is able to block the entrance and propagation of α-syn in neurons, and hence prevents the development of neuropathological abnormalities in the brain (Tran et al., 2014).

However, several important questions remain to be solved (**Table 2**): (i) it is currently unknown whether the pathological conversion of endogenous α-syn triggered by PD-derived material or recombinant α-syn fibrils actually occurs directly through a seeding process or indirectly as a general response to cellular stress; (ii) the association between pathological α-syn accumulation and neuron cell death remains so far correlative. In addition, there is no definitive evidence to support the idea that PD can be contagious from one person to another, as is characterized for prion diseases (Beekes et al., 2014). In this line, a retrospective, postmortem study of recipients of cadaver-derived human growth hormone (hGH) found no reported incidence of PD, although the donors of pituitary glands used for hGH preparation probably included people with PD, and pathological α-syn is frequently found in the postmortem pituitary glands of people with PD (Irwin et al., 2013). One of the possible experiments would be to isolate α-syn aggregates developed in PD-derived material or recombinant α-syn fibrils injected animals, and injecting again in a healthy animals. These experiments would allow us to differentiate between infectious and self-propagating properties. Some approaches should be tested to evaluate the transmission of these disorders between animals (mice and monkeys) in order to study species-barrier properties or the use of different administration routes (intracerebrally, intranasal or fluids). All these studies should answer the unavoidable question of infectivity and/or contagiousness, the last missing criterion that defines a prion disease. However, until the issues mentioned above around nature and mechanisms of α-syn prion-like properties are better understood, we believe that the term prion for α-syn has to be used and considered cautiously. A new term referring as self-propagating pathogenic protein for α-syn needs to emerge and this is a mechanism well worth considering.

## Conflict of Interest Statement

The authors declare that the research was conducted in the absence of any commercial or financial relationships that could be construed as a potential conflict of interest.
